# Contrasting Environmental Drivers Determine Biodiversity Patterns in Epiphytic Lichen Communities along a European Gradient

**DOI:** 10.3390/microorganisms8121913

**Published:** 2020-12-01

**Authors:** Pilar Hurtado, María Prieto, Francesco de Bello, Gregorio Aragón, Jesús López-Angulo, Paolo Giordani, Eva María Díaz-Peña, Rebeca Vicente, Sonia Merinero, Alica Košuthová, Renato Benesperi, Elisabetta Bianchi, Helmut Mayrhofer, Juri Nascimbene, Martin Grube, Mats Wedin, Martin Westberg, Isabel Martínez

**Affiliations:** 1 Área de Biodiversidad y Conservación, ESCET, Universidad Rey Juan Carlos, c/Tulipán s/n, 28933 Móstoles, Spain; maria.prieto@urjc.es (M.P.); gregorio.aragon@urjc.es (G.A.); jesus.lopez.angulo@urjc.es (J.L.-A.); evamaria.diazp@gmail.com (E.M.D.-P.); r.vicentemurjc@gmail.com (R.V.); sonia.merinero@gmail.com (S.M.); isabel.martinez@urjc.es (I.M.); 2 Centro de Investigaciones sobre Desertificación (CSIC-UV-GV), Carretera Moncada-Náquera km. 4.5, 46113 Valencia, Spain; Francesco.Bello@ext.uv.es; 3 Department of Botany, Faculty of Sciences, University of South Bohemia, Branišovská 1760, 370 05 České Budějovice, Czech Republic; 4 DIFAR, University of Genova, Viale Cembrano, 4, I-16148 Genova, Italy; giordani@difar.unige.it; 5 Department of Ecology, Swedish University of Agricultural Sciences, P.O. Box 7044, SE-750 07 Uppsala, Sweden; 6 Department of Botany, Swedish Museum of Natural History, P.O. Box 50007, SE-104 05 Stockholm, Sweden; alica.kosuthova@nrm.se (A.K.); Mats.Wedin@nrm.se (M.W.); 7 Department of Botany and Zoology, Faculty of Science, Masaryk University, 611 37 Brno, Czech Republic; 8 Department of Biology, University of Florence, Via La Pira, 4, I-50121 Firenze, Italy; renato.benesperi@unifi.it (R.B.); e.bianchi@unifi.it (E.B.); 9 Institute of Biology, Karl-Franzens-Universität Graz, Holteigasse 6, 8010 Graz, Austria; helmut.mayrhofer@uni-graz.at (H.M.); martin.grube@uni-graz.at (M.G.); 10 Department of Biological, Geological and Environmental Sciences, University of Bologna, Via Irnerio, 42, I-40126 Bologna, Italy; juri.nascimbene@unibo.it; 11 Museum of Evolution, Uppsala University, Norbyvägen 16, SE-752 36 Uppsala, Sweden; martin.westberg@em.uu.se

**Keywords:** beech forests, climate, epiphytic lichen, functional diversity, functional trait, latitudinal gradient, phylogenetic diversity, taxonomic diversity

## Abstract

Assessing the ecological impacts of environmental change on biological communities requires knowledge of the factors driving the spatial patterns of the three diversity facets along extensive environmental gradients. We quantified the taxonomic (TD), functional (FD), and phylogenetic diversity (PD) of lichen epiphytic communities in 23 beech forests along Europe to examine their response to environmental variation (climate, habitat quality, spatial predictors) at a continental geographic scale. We selected six traits related to the climatic conditions in forest ecosystems, the water-use strategy and the nutrient uptake, and we built a phylogenetic tree based on four molecular markers. FD and climate determined TD and PD, with spatial variables also affecting PD. The three diversity facets were primarily shaped by distinct critical predictors, with the temperature diurnal range affecting FD and PD, and precipitation of the wettest month determining TD. Our results emphasize the value of FD for explaining part of TD and PD variation in lichen communities at a broad geographic scale, while highlighting that these diversity facets provide complementary information about the communities’ response under changing environmental conditions. Furthermore, traits such as growth form, photobiont type, and reproductive strategy mediated the response of lichen communities to abiotic factors emerging as useful indicators of macroclimatic variations.

## 1. Introduction

Unveiling the factors that drive the heterogeneous distribution of biodiversity at a broad geographical scale is a primary goal to predict the response of communities to environmental change [1]. Community ecologists have largely evaluated how the spatial differences in biodiversity have been generated, identifying climate and primary productivity as some of the factors underpinning patterns such as the latitudinal diversity gradient [2]. Although the knowledge on biodiversity patterns traditionally relied on species composition or richness, the few studies that integrate taxonomic (TD), functional (FD), and phylogenetic (PD) diversity facets prove that they show different responses to environmental changes [3,4]. These findings highlight the limitations of a purely taxon-based approach to assess the impact of environmental changes on biodiversity [5]. It is, thus, needed to consider the response of FD and PD because different species within the communities represent distinct functions and evolutionary histories [6]. Therefore, the patterns of biodiversity shaped by the environmental conditions may differ depending on which species, traits, or lineages within a community are affected [7].

Since biodiversity patterns can differ substantially depending on the facet considered, an effective assessment of the communities’ response requires knowledge of the relationships between TD, FD, and PD [8]. The extent to which these three facets are related along broad-scale gradients is still unclear, especially for some historically-overlooked elements of biodiversity such as lichens. For example, it is expected that TD is positively related to FD and PD based on the assumption that a higher number of species represent a higher range of trait values and lineages [9]. However, a decrease in TD does not necessarily imply a decrease in FD and PD if functionally redundant species are removed [10,11] or if certain clades are overrepresented within the community [12,13]. Furthermore, FD and PD might be correlated if the functional traits mediating the persistence of species within a community display a significative phylogenetic signal, meaning that close relatives exhibit more similar values for those traits than distant relatives [14]. Studies assessing the relationships between TD, FD, and PD have found inconsistent results [8,9], which highlights the importance of disentangling the role of these diversity facets as surrogates for the others under contrasting environmental conditions.

The key to understand TD, FD, and PD patterns and predict the potential community changes, is to disentangle the specific drivers shaping these facets of biodiversity along extensive gradients. Overall TD, FD, and PD trends can be determined by different predictors, which modify different diversity facets in different ways [15]. Thus, TD, FD, and PD might respond differently to environmental factors. For example, Chun and Lee [16] found that climate was the main driver of PD in plant communities along an altitudinal gradient in East Asia, while habitat heterogeneity mainly determined FD. Accordingly, in a global scale study focused on mammals, Safi et al. [17] found that PD was influenced by the mean annual temperature, while FD was modulated by seasonality. Thus, if these diversity facets are not always correlated and represent independent aspects of community structure, it is important to find which predictors modify them.

In this study we examine the biodiversity patterns in response to a continent-wide climatic, latitudinal, and longitudinal gradient for addressing: (1) what the contribution is of environmental drivers determining TD, FD, and PD, and what are the relationships between them, in lichen communities along a wide latitudinal gradient; (2) whether there is a geographical structuration of the species and trait composition of the communities in response to the environmental conditions. To address these questions, we quantified TD, FD, and PD of lichen epiphytic communities in 23 beech forests from northern to southern Europe (circa 3000 km). We focused on lichens because these organisms lack active mechanisms to regulate nutrient and water uptake and loss and, consequently, they are physiologically active, strictly depending on the environmental conditions [18]. Therefore, they are very sensitive indicators of environmental changes [19]. We selected three qualitative functional traits (growth form, photobiont type, and reproductive strategy), which have been related to the climatic conditions in forest ecosystems [20]. Moreover, we quantified three physiological traits related to the water-use strategy (specific thallus mass, STM, and water-holding capacity, WHC) and nutrient uptake (carbon-nitrogen ratio, C/N). We hypothesize that distinct climatic drivers determine TD, FD, and PD patterns, which might be related to each other. We expect that lichen communities respond to environmental conditions through their functional traits and, thus, FD modulates TD and PD [3,4]. However, we do not expect a relation between TD and PD [3].

## 2. Materials and Methods

### 2.1. Study Area and Sampling Design

**Figure 1:**
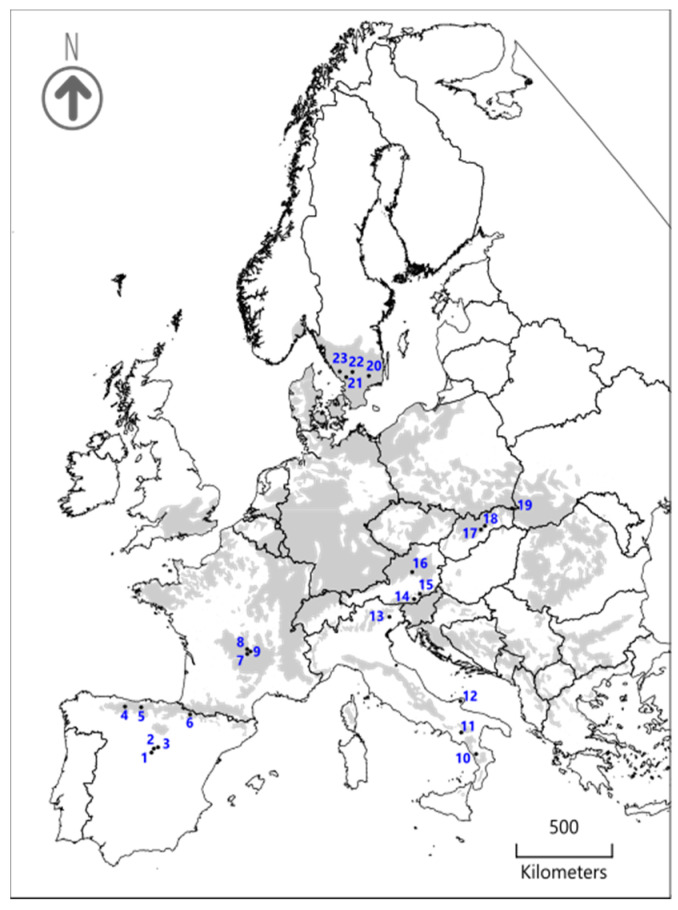
Map of the study area showing the distributional range of *Fagus sylvatica* (light grey) and the sampling sites (numbers and black dots). Name of the sampling sites in Table S1.

The study area encompassed six European countries located along a latitudinal gradient from 57° N (Sweden) to 39° S (Italy), and a longitudinal gradient from 5° W (Spain) to 22° E (Slovakia). The gradient is characterized by strong climatic differences in mean annual temperature (from 3.9 to 11.9 °C) and total annual precipitation (from 563 to 1644 mm), as well as in temperature and precipitation seasonality (Table S1) [21]. We surveyed lichen epiphytic communities in 23 mature and well-conserved monospecific stands of European beech (i.e., tree cover >65% and 50 years without tree cutting) along a latitudinal and longitudinal gradient covering the entire range of *Fagus sylvatica* L. (Figure 1). To minimize the effect of sampling communities in different successional stages, we only surveyed beech stands with the lichen species *Lobaria pulmonaria* (L.) Hoffm.

We surveyed the composition of lichen communities following Aragón et al. [22]. Within each stand, we randomly established five 25 × 25 m plots with a minimum distance to forest edge of 100 m and a minimum distance of 500 m among plots. Within each plot, we selected 10 beech trees with a minimum diameter at breast height (DBH) >25 cm. On each tree, we established four 20 × 30 cm grids on the trunk, at two different aspects (north and south faces) and heights (breast and tree base). Within each grid, we recorded lichen cover (%). In total, we surveyed 1150 trees and collected data from 4600 grids. For quantitative functional trait measurements, we collected a maximum of four thalli of all macrolichen species per forest stand. Samples were air-dried and stored at −20 °C before trait measurements in the laboratory.

### 2.2. Trait Data and Phylogenetic Tree

We categorized all surveyed species according to three qualitative traits: growth form, photobiont type, and reproductive strategy (Table S2). These are easily discernible traits broadly used to differentiate lichen functional groups concerning water uptake and loss, response to climate, or colonization and establishment [23,24]. We followed the ITALIC database [25] and the Lias light database [26] for qualitative trait classification. Growth form included seven categories (crustose, squamulose, leprose, foliose broad lobed, foliose narrow lobed, fruticose dorsiventral, and fruticose filamentous), while both photobiont type (green algae, *Trentepohlia*, and cyanobacteria) and reproductive strategy (sexual, asexual, and both) included three categories.

We measured three quantitative traits related to water-use strategy (n = 1018 thalli) and nutrient uptake (n = 1179 thalli) for macrolichens (Table S2). Regarding water-use strategy, we measured specific thallus mass (STM) and water holding capacity (WHC) according to Merinero et al. [27]. For nutrient uptake, we quantified thallus carbon-nitrogen ratio (C/N) using a PDZ Europa ANCA-GSL elemental analyzer interfaced to a PDZ Europa 20–20 isotope ratio mass spectrometer (Sercon Ltd., Cheshire, UK) at UC Davis Stable Isotope Facility.

We built a multigene phylogenetic tree for four molecular markers (nuITS, nuLSU, mtSSU, and RPB1) using sequences from GenBank or generating the sequences following Prieto and Wedin [28] for those species not available in this database (see [24] for further details about the phylogenetic analysis) (Figure S1). These molecular markers were selected based on previous studies, which obtained supported relationships at different hierarchical levels [4,28]. We tested the phylogenetic signal for the three categorical traits with the function *phylo.signal.disc* developed by Enrico Rezende in the *ape* R package. A significant phylogenetic signal (*p* < 0.05) was detected when the observed trait change rates were lower than the ones expected by chance. For quantitative traits, we calculated the phylogenetic signal (Pagel’s Lambda) using the function *phylosig* in *phytools* R package.

### 2.3. Diversity Metrics

We used species composition and cover, and trait and phylogenetic information to calculate taxonomic, functional, and phylogenetic diversity indices at the community level including the 23 forest stands surveyed. To do so, we merged all data within each forest stand, averaging species covers across all samples within a stand. We calculated three complementary taxonomic diversity indices (i.e., lichen species richness, Shannon, and Inverse Simpson) for each forest stand because we expected environmental effects on the presence and abundance of the species. Species richness refers to the number of species, while both Shannon and Inverse Simpson are weighted by species abundances, with Inverse Simpson better capturing species dominance. Regarding the functional characterization of the epiphytic communities, we used individual species trait data to calculate two indices at the community level: community weighted mean (‘CWM’) and Rao’s quadratic entropy index (‘Rao’). Finally, we calculated a Rao index combining the species relative abundance with the phylogenetic tree as a metric of PD (see Supplementary 4 for further information about diversity metrics).

### 2.4. Environmental Drivers

We assessed the response of taxonomic, functional, and phylogenetic community diversity to a set of variables related to spatial information, forest structure and climate. For spatial information, we recorded the longitude, latitude, and altitude for all the 23 beech forests studied. We measured the tree diameter at breast height (DBH) of the 50 trees sampled within each forest and calculated the mean DBH as a proxy of forest structure. Climatic information at the forest level was retrieved from the high-resolution climate database CHELSA [21] including 19 bioclimatic variables related to temperature and precipitation (Table S1).

### 2.5. Data Analyses

#### 2.5.1. Environmental Driver Contribution and Relationships between TD, FD, and PD Determining Biodiversity Patterns across Europe

First, we analyzed the relationships between TD with FD and PD and between PD and FD using Linear Models (LMs). We used a multi-model inference approach to identify the subset of models with stronger empirical support [29]. We included linear and quadratic terms to allow for the curvilinear effects of the explanatory variables. Based on the second-order Akaike Information Criterion (AICc) for small sample sizes, we compared the AICc of the alternative models with the AICc of the best candidate model (lowest AICc) and we selected all the models with ΔAICc < 2. Variance explained (R^2^) was calculated for each model to estimate the explanatory value. For the set of selected models, we quantified the relative likelihood of each model (Akaike weights, wi), and the relative importance (w+) of each explanatory variable by summing the Akaike weights (wi) of all models in which the predictor was selected and considering the number of models in which this predictor appears [29]. In all cases, QQ-plots and predicted against residual plots were used to verify model assumptions [30]. Moran’s Index was used to test for spatial autocorrelation in the residuals of the selected models with the function *Moran. I* in the R package *ape*. When residuals of a model showed significant spatial autocorrelation (*p* < 0.05), we added the coordinates of each plot, the quadratic terms, and the interaction term to the LM. Analyses were performed with *MASS*, *vegan*, and *MuMIn* R packages.

Second, we evaluated the effect of environmental drivers on the community diversity metrics using LMs. To avoid multicollinearity and to reduce the number of explanatory variables, we estimated the variance inflation factor (VIF) and we excluded highly correlated predictors according to Pearson correlation coefficients (r > 0.7, *p* < 0.05), selecting those predictors which were a priori more ecologically relevant for lichens’ biology. The resulting set of explanatory variables included in the models were annual mean diurnal range, maximum temperature of the warmest month, minimum temperature of the coldest month, mean temperature of the wettest quarter, precipitation of the wettest month, precipitation of the driest month, and DBH. We built separate models using each diversity index as dependent variables and the selected climatic and forest structure variables as independent variables. We used a multi-model inference approach and selected the best models (ΔAICc < 2) as explained above.

Third, we applied a variation partitioning analysis [31] to evaluate the unique and shared fractions of variation of TD explained by FD, PD, and the environmental drivers, and the unique and shared fractions of variation of PD explained by FD and the environmental drivers. Variation partitioning was applied (*varpart* in *vegan* R package) based on the adjusted R^2^ statistics derived from the previous LMs, considering only the predictors with relative importance (w+) > 0.4 [32]. When residuals of a model showed significant spatial autocorrelation, we added to the variation partitioning the spatial variable selected by the AICc procedure to evaluate the shared effect with the explanatory variables on TD and PD.

#### 2.5.2. Contribution of Climatic Drivers Determining Species Composition and Functional Structure of Lichen Communities across Europe

We used constrained ordination analyses to evaluate the multivariate relationships between all the 19 climatic drivers and the changes in community species composition and CWM of growth form, photobiont type, and reproductive strategy [33]. To decide whether to apply linear or unimodal ordination methods, we first conducted a Detrended Correspondence Analysis (DCA) with the species composition and the CWM matrices separately. We concluded that linear methods were suitable since the length of the first DCA axis < 3 standard deviation units [34]. For species composition and CWM, we performed two separate Redundancy Analyses (RDAs), which is a constrained ordination method that assumes linear responses of dependent variables with the extracted axes. We first tested the significance of the global models (including all the explanatory variables) with a Monte Carlo permutation test (999 permutations). Given that the global RDA models were significant (species composition: F-ratio statistics = 2.109, *p* = 0.03; CWM: F-ratio statistics = 4.499, *p* = 0.004), we applied a forward stepwise procedure to select a subset of explanatory variables, maintaining the variation explained by them to the maximum. We followed two different approaches to select the explanatory variables, one based on Monte Carlo permutation tests (function *ordistep* in *vegan* R package) and the other based on adjusted R^2^ (R^2^adj) (function *ordiR2step* in the *vegan* R package) [35]. We finally conducted an RDA with the following selected explanatory variables: isothermality and precipitation seasonality for community species composition, and annual mean temperature, isothermality, annual precipitation, and precipitation of the warmest quarter for CWM of qualitative traits.

We then applied a variation partitioning analysis (function *varpart* in the *vegan* R package) to evaluate the unique and shared fractions of species composition and CWM variation explained by the climatic drivers. Variation partitioning was applied based on the adjusted R^2^ statistics considering only the explanatory variables forward-selected by the previous RDA analyses.

## 3. Results

### 3.1. Environmental Driver Contribution and the Relationships between TD, FD, and PD Determining the Biodiversity Patterns across Europe

Both TD and PD were related to FD, but TD and PD were not related to each other (Table 1). The relationship between TD and FD was unimodal with a trend of increasing TD as FD increases, but with a clear decrease in TD for high FD values (Figure S2). PD increased with increasing FD (Figure S2). Overall, the percentage of variance explained by each model was circa 30% and none of the best-fit models showed significant spatial autocorrelation, except the model relating PD and FD (Table 1). We found a high phylogenetic signal for all quantitative and qualitative traits (Table S3).

**Figure 2:**
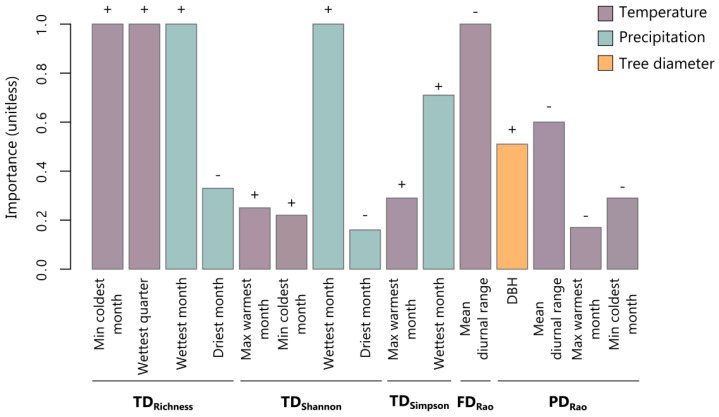
Relative importance (w+) of climate and landscape structure in linear models of community diversity metrics. Bar height represents the relative importance of each predictor quantified as the sum of Akaike weights (wi) of all models in which the predictor was selected and considering the number of models in which this predictor appears. Positive (+) or negative (−) effects of each predictor on a given diversity metric are indicated above the bar. Abbreviations: taxonomic (TD), functional (FD), and phylogenetic (PD) diversity; Inverse Simpson index (Simpson); tree diameter (DBH).

The three diversity facets responded to environmental predictors, but the most important drivers behind this response differed for TD, FD, and PD (Figure 2, Table S4). While temperature variables significantly influenced all the three facets of biodiversity, TD and PD also responded to precipitation and tree diameter changes, respectively (Figure 2, Table S4). Annual mean diurnal range was the most important predictor of FD and PD: these two diversity facets decreased with increasing temperature fluctuation over the year (Figure 2). Additionally, PD increased with increasing tree diameter and, to a lesser extent (w+ < 0.4), with decreases in maximum temperature of the warmest month and minimum temperature of the coldest month (Figure 2). Contrary to FD and PD, species richness was mainly determined by higher minimum temperature of the coldest month, mean temperature of the wettest quarter, and precipitation of the wettest month (Figure 2). Therefore, oceanic sites hosted communities with a higher number of species. Communities with higher Shannon and Inverse Simpson were associated with areas with higher wettest month precipitation (Figure 2).

**Figure 3:**
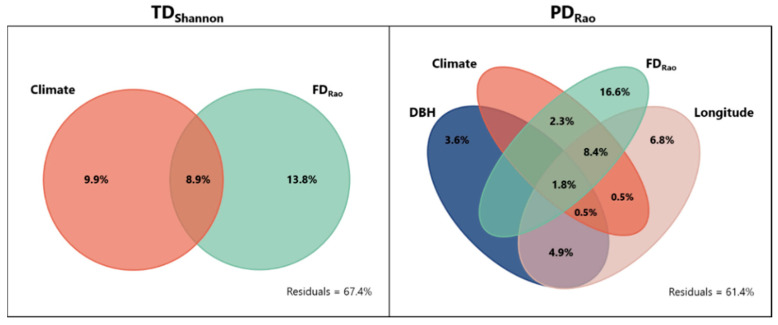
Variation partitioning Venn diagrams representing the unique and shared contribution (%) of climate and FD to TD variation (**left**), and DBH, climate, FD, and longitude to PD variation (**right**). The intersections represent the percentage of explained variation shared by different explanatory variables. Areas without values represent 0 or negative percentages of explained variance. Residuals represent the percentage of unexplained variation.

Considering the results of the LMs analyzing both the relation between TD, FD, and PD (Table 1), and the effect of environmental variables on such diversity facets (Figure 2), we performed variation partitioning analyses to evaluate the unique and shared fractions of TD variation explained by climate and FD, and the unique and shared fractions of PD variation explained by DBH, climate, FD, and longitude (Figure 3). We selected Shannon as a metric for TD because the model with Shannon had a higher R^2^ value than models of Inverse Simpson and because species richness was not related with FD and PD (Table 1) (in any case, results for Inverse Simpson can be found in Figure S3). Precipitation of the wettest month and FD explained circa 33% of TD variation. FD was the main contributor, explaining most of such variation (circa 14%), and almost half of the variation in Shannon explained by climate was shared with FD (Figure 3). For PD, almost 40% of variation was explained by DBH, annual mean diurnal range, FD, and longitude. The main predictor was FD, both in their unique (circa 17%) and shared fractions with DBH, climate, and longitude (Figure 3). After FD, longitude (6.8%) and DBH (3.6%) showed the highest unique fractions explaining PD. All the contribution of climate explaining PD was shared, mainly with FD and longitude, and, to a lesser extent, with DBH (Figure 3).

### 3.2. Contribution of Climatic Drivers Determining Species Composition and Functional Structure of Lichen Communities across Europe

**Figure 4:**
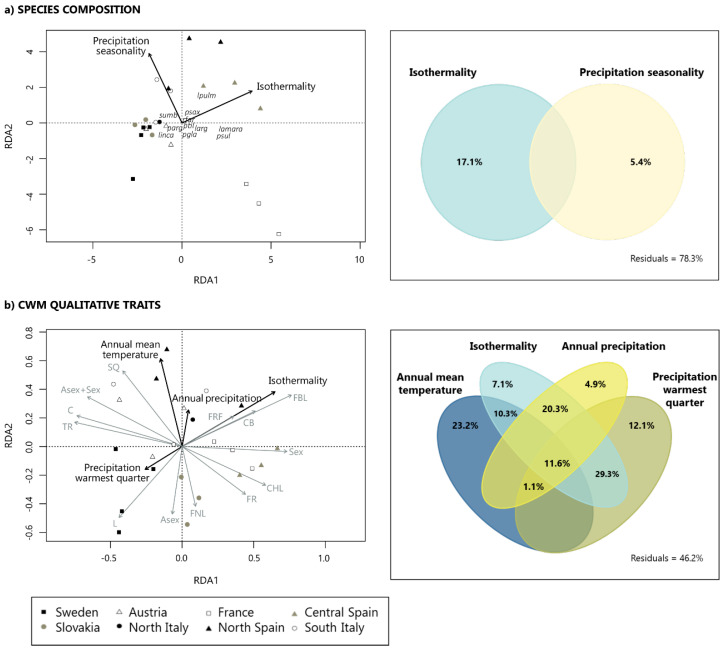
First two axes of the redundancy analysis ordinations (RDAs) and variation partitioning Venn diagrams representing the amount (%) of explained variation shared by different explanatory variables (intersection), and the percentage of the unexplained variation (residuals). Areas without values represent 0 or negative percentages of explained variance. (**a**) For species composition, the first two RDA axes explained 28.81% of the variance. (**b**) For CWM of qualitative traits, the first two RDA axes explained 62.19% of the variance. Only the climatic variables that significantly explained variability in lichen epiphytic community composition (**a**) and CWMs (**b**) following the forward stepwise procedure are shown (black arrows). Each point represents the lichen community at a specific forest (*n* = 23) and the labels in italic denote the species with a higher weight on the ordination axis. Each grey arrow represents the CWM of the qualitative trait categories. The arrows direction indicates the maximum change of that variable, and the length is proportional to the rate of change. Abbreviations: (1) Species: larg = *Lecanora argentata*; lamara = *Pertusaria amara*; linca = *Lepraria incana*; lpulm = *Lobaria pulmonaria*; psax = *Parmelia saxatilis*; psul = *Parmelia sulcata*; ptil = *Parmelina tiliacea*; parg = *Phylctis argena*; pgla = *Platismatia glauca*; rfar = *Ramalina farinacea*; sumb = *Scoliciosporum umbrinum*; (2) Traits: for growth form: C = crustose, SQ = squamulose, L = leprose, FBL = foliose broad lobed, FNL = foliose narrow lobed, FR = fruticose, FRF = fruticose filamentous; for photobiont: CB = cyanolichen, CHL = chlorolichen, TR = *Trentepohlia*; for reproduction: Asex = asexual, Sex = sexual, Asex + Sex = with asexual and sexual reproduction.

RDA models were significant for species composition (F-ratio statistics = 4.046, *p* = 0.001) (Figure 4a) and CWM of growth form, photobiont type, and reproductive strategy (F-ratio statistics = 7.4002, *p* = 0.001) (Figure 4b). Species composition and functional structure of lichen communities were influenced by different climatic variables (Figure 4).

For species composition, isothermality (daily temperature range divided by the annual temperature range) and precipitation seasonality explained the variation in the taxonomic composition of lichen communities along the gradient. These climatic variables explained circa 22% of species composition variation, with a more important contribution of isothermality (17.1%) than precipitation seasonality (5.4%) (Figure 4a). Lichen communities from areas with higher temperature fluctuations within a month (France and Spain) differed from those with smaller temperature fluctuations (Sweden, Slovakia, Austria, and northern Italy). We also identified a gradient in precipitation seasonality, isolating lichen communities of France (with the lowest values of precipitation seasonality) from the rest (Figure 4a).

CWM of growth form, photobiont type, and reproductive strategy responded to temperature (annual mean temperature and isothermality) and precipitation variables (annual precipitation and precipitation of the warmest quarter) (Figure 4b). These four variables explained circa 54% of the variation, with the annual mean temperature (23.2%) and precipitation of the warmest quarter (12.1%) showing the highest unique contributions (Figure 4b). Crustose and squamulose species and those lichens with two types of reproductive strategies were mainly associated with sites with higher annual mean temperature (Figure 4b). In contrast, foliose narrow lobed, fruticose, leprose, chlorolichens, and species with asexual reproduction were associated with colder sites (Figure 4b). Moreover, foliose broad lobed, fruticose filamentous and cyanolichens thrived in sites with high isothermality and low precipitation during the warmest quarter, which are climatic conditions characteristic of the southern forests (Figure 4b).

## 4. Discussion

Functional diversity, climate, and landscape structure determined the patterns of taxonomic and phylogenetic diversity in lichen communities across Europe, with spatial variables (longitude) also determining the variation in phylogenetic diversity. Throughout the gradient, an increasing FD correlated with an increasing TD and PD, pointing out functional complementarity and a strong phylogenetic signal for the studied traits. Although FD explained part of the variation in TD and PD, these two latter facets were not related, supporting the assumption that they provide complementary information about the response of the communities [5]. Interestingly, the three diversity facets were primarily shaped by distinct environmental predictors, indicating that a multidimensional approach is needed to unveil biodiversity patterns in response to environmental changes. The geographic variation of FD and PD along this 3000 km latitudinal gradient responded to changes in temperature diurnal range, with tree diameter and longitude also affecting PD, while patterns of TD were overall determined by precipitation of the wettest month. However, biotic constrains and different abiotic factors to those analyzed in this study may also drive the spatial patterns of lichen biodiversity across Europe. Climate explained a large amount of variation (54%) in the studied functional traits, highlighting their role as indicators of climatic changes. Our results indicated the value of the studied functional traits to infer the effects of abiotic drivers and understand the mechanisms that structure lichen communities, suggesting that species enter and persist in natural communities based on their functional trait values [36,37].

Here, we show that climate and FD modulated the patterns of TD, with a higher contribution of FD. Almost half of the effect of climate over TD was shared with FD, suggesting that climatic factors might act as abiotic filters affecting species through their functional traits [36,37]. Previous studies demonstrated that environmental gradients affected all taxonomic, functional, and phylogenetic diversity patterns [4,16,17]. However, the major drivers shaping such patterns varied depending on the facet considered, even though some of these diversity facets were related [3]. Lichen communities with high FD tended to display a high TD reflecting high functional complementarity [3,4,38]. Thus, different species within these communities may have different roles, being more resistant and resilient to variable environmental conditions [39]. However, we found a clear decrease in TD for the highest values of FD: three lichen species with different traits represented circa 60% of the total cover in communities located in two of the northernmost forests (Sweden). Therefore, these communities are highly uneven, dominated by a few species with very different functional strategies, likely enhancing their resistance under these precise climatic conditions and probably reflecting a process of limiting similarity in these northernmost forests [40,41].

FD was the major driver modulating PD variation along the gradient, but spatial variables, climate, and tree diameter also contributed. The positive relation between FD and PD might reflect the strong phylogenetic signal of the studied traits, meaning that communities composed of distant relatives display a wider range of trait values [42]. The lack of relation between PD and TD might respond to the existence of mechanisms selecting for clades of closely related species within the studied communities [13,43]. Interestingly, all the contribution of climate to PD variation was shared with FD and the spatial variables. Contrary to the results obtained for macrolichens [4], increasing temperature fluctuation during the day resulted in functional and phylogenetic homogenization of lichen communities, suggesting that only species with a certain range of functional traits, which provides a better acclimation strategy to temporal changes in temperature, will persist. Under marked temperature fluctuations, assemblages were composed of close relatives with similar traits, indicating a high trait redundancy under these conditions. These results suggest that the main climatic factors determining biodiversity patterns not only differ depending on the biodiversity facet, but also on the organisms considered.

Changes in precipitation during the wettest month and temperature factors did shape patterns of TD, pointing at the poikilohydric nature of lichens. Oceanic sites, with warmer and more humid conditions, favored an increase in the number and diversity of species within the communities [4]. Tree diameter, which reflects the amount of space and time available for colonization and which is an indicator of habitat quality in epiphytic lichens, only affected PD but not FD and TD [44]. In sites with larger trees, there was a replacement of species with similar traits (i.e., TD and FD do not vary), but with distinct evolutionary or biogeographical histories (i.e., high PD). This result suggests a parallel evolution of traits within the communities, meaning that species with different origins share functional traits, as has been previously demonstrated in lichens (e.g., [45]). Finally, we found a longitudinal trend with PD increasing towards eastern Europe, but most of the contribution of longitude to PD variation was shared with climate, suggesting the existence of a climatic gradient spatially structured across the study area. Longitude also explained a unique fraction of variation in PD, which could be related to the post-glacial expansion of *Fagus sylvatica* from multiple scattered Pleistocene refugia in Europe [46]. Beech populations in southern Europe increased slowly and moderately, and are currently declining and, with them, the presence of ancient lichen lineages within the communities. In contrast, in central Europe, beech populations showed a quick and extensive increase, reaching northern Europe [46].

To better understand the observed patterns of biodiversity in response to climatic gradients, we analyzed the changes in species composition and functional structure of the lichen communities. We identified a geographical structuration of species composition of the epiphytic communities along with the temperature and precipitation seasonality gradients. As reported by Matos et al. [19] for functional traits, we found that temperature and seasonality, rather than precipitation and mean values, are the main drivers determining species composition in lichen communities. In both cases, the communities located at the extremes of these climatic gradients corresponded to the southernmost forests (i.e., France, Spain, and Italy), which were clearly differentiated from those communities located in the northern sites. The climatic factors accounted for 22% of the species composition variability, whereas almost 54% of the community-level trait variation was explained by climate. These results support the idea that species thriving in natural communities respond to climatic changes through their functional traits [36,47] and emphasize the value of the selected suite of qualitative traits as ecological indicators [20]. As observed for species composition, and consistent with patterns in FD, changes in CWMs of growth form, photobiont type, and reproductive strategy also responded to temperature rather than precipitation variables. Lichens are only intermittently active during certain seasonal cycles and can acclimate their physiological activity to light and temperature changes [48]. Our results indicate that temperature fluctuations constrain the metabolic rates of lichen species rather than precipitation changes along the studied gradient. Temperature-related variables act as filters selecting for certain traits that do perform better under these constraints (e.g., crustose and squamulose species at higher mean annual temperatures, fruticose and asexual species at low mean annual temperatures, or cyanolichens at sites with high isothermality).

In conclusion, we found partial congruence between taxonomic, functional, and phylogenetic diversity patterns in lichen communities across Europe, suggesting that FD could be considered a good proxy for TD and PD. However, these latter two mismatched, and distinct critical environmental predictors determined TD, FD, and PD patterns. These results emphasize the need to adopt a multidimensional approach explicitly incorporating measures of TD, FD, and PD to assess the response of lichen communities to environmental changes. We found high functional complementarity and a strong phylogenetic signal for a suite of traits related to water and nutrient uptake and loss, response to climate, colonization, and establishment. Our results suggest that epiphytic lichens enter and persist in natural communities based on their qualitative functional traits (growth form, photobiont type, and reproductive strategy), which appeared as valuable indicators of global change drivers such as macroclimatic changes.

## Figures and Tables

**Table 1:** Ranking of linear models analyzing the relationship between TD, FD, and PD following an AIC-based model selection procedure. For each community diversity metric, the best model (lowest AICc value) is presented in the first row followed by the rest of models with ΔAICc ≤ 2. Grey cells indicate predictors included in a particular model. Only significant relationships (*p* < 0.05) are shown. R^2^adj, percentage of variance explained by a particular model; AICc, relative goodness of fit; ΔAICc, AIC differences; wi, Akaike weight; Ip, Moran index *p*-value.

	FD_Rao_		PD_Rao_						
	Linear	Quadratic	Linear	Quadratic	R^2^	AICc	ΔAICc	wi	Ip
TD_Richness_					0	180.1	0	1	-
TD_Shannon_					0.30	−3	0	1	0.58
TD_Inverse Simpson_					0.27	104.6	0	0.68	0.61
					0	106.1	1.52	0.32	-
PD_Rao_			-	-	0.29	−20	0	1	0.004 *

* Asterisk indicates significant spatial autocorrelation according to Moran index.

## Supplementary Materials

The following are available online at https://www.mdpi.com/2076-2607/8/12/1913/s1, Table S1: Environmental variables characterizing the 23 beech forests studied, Table S2: Functional trait values, Figure S1: Phylogenetic tree, Table S3: Phylogenetic signal, Supplementary 4: Diversity metrics, Figure S2: Relationships among TD, FD, and PD, Table S4: Ranking of linear models of species richness, Shannon, Inverse Simpson, Rao FD (multitrait) and Rao PD, Figure S3: Variation partitioning Venn diagram for Inverse Simpson.
