# Supplementary material for: Contrasting Environmental Drivers Determine Biodiversity Patterns in Epiphytic Lichen Communities along a European Gradient
Source: Microorganisms. 2020 Dec 1;8(12):1913. doi: 10.3390/microorganisms8121913 (PMC7760525; doi:10.3390/microorganisms8121913)
Supplement: Supplementary file 1 [file microorganisms-08-01913-s001.pdf]

**Table 1.** Environmental variables characterizing the 23 beech forests studied.

| | | | | | CLIMATIC VARIABLES | | | | | | | | | | | | | | | | | | |
|---|---|---|---|---|---|---|---|---|---|---|---|---|---|---|---|---|---|---|---|---|---|---|---|
| | | | | | **Temperature** (°C) | | | | | | | | | | | **Precipitation** (mm) | | | | | | | |
| **Sampling site** | **Latitude** (degrees) | **Longitude** (degrees) | **Altitude** (m a.s.l.) | **DBH** (m) | **Annual Mean** | **Mean diurnal range** | **Isothermality** (unitless) | **Seasonality** (unitless) | **Max warmest month** | **Min coldest month** | **Annual range** | **Mean wettest quarter** | **Mean driest quarter** | **Mean warmest quarter** | **Mean coldest quarter** | **Annual** | **Wettest month** | **Driest month** | **Seasonality** (unitless) | **Wettest quarter** | **Driest quarter** | **Warmest quarter** | **Coldest Quarter** |
| sw_oder (23) | 57.0 | 13.5 | 197.0 | 0.57 | 6.7 | 65.0 | 255 | 6502 | 20.6 | -4.9 | 25.5 | 4.3 | 3.8 | 15.8 | -1.8 | 914 | 95 | 50 | 22.0 | 281 | 162 | 266 | 179 |
| sw_ramlak (22) | 57.1 | 12.6 | 107.8 | 0.50 | 7.5 | 49.6 | 214 | 6309 | 20.0 | -3.2 | 23.2 | 5.4 | 4.4 | 16.4 | -0.8 | 842 | 90 | 44 | 23.8 | 267 | 148 | 251 | 156 |
| sw_bisk (21) | 56.8 | 12.9 | 141.6 | 0.65 | 7.0 | 57.0 | 236 | 6354 | 20.0 | -4.1 | 24.1 | 4.8 | 4.0 | 15.9 | -1.4 | 1069 | 117 | 57 | 23.0 | 335 | 188 | 328 | 203 |
| sw_bjur (20) | 56.6 | 14.7 | 161.0 | 0.40 | 6.9 | 62.0 | 248 | 6489 | 20.8 | -4.4 | 25.2 | 15.8 | 3.1 | 16.1 | -1.5 | 658 | 76 | 37 | 22.0 | 211 | 115 | 203 | 125 |
| sk_rabia (19) | 49.10 | 22.5 | 1151.4 | 0.68 | 4.0 | 73.0 | 259 | 7484 | 18.7 | -9.6 | 28.3 | 13.7 | -6.0 | 13.9 | -6.2 | 1125 | 144 | 60 | 29.8 | 430 | 182 | 368 | 213 |
| sk_mp (18) | 48.76 | 20.1 | 900.2 | 0.34 | 6.2 | 75.2 | 261 | 7581 | 21.2 | -7.7 | 29.0 | 13.8 | -3.7 | 16.3 | -4.1 | 666 | 87 | 32 | 30.2 | 250 | 101 | 201 | 106 |
| sk_kv (17) | 48.69 | 19.8 | 1233.4 | 0.59 | 3.9 | 76.0 | 264 | 7439 | 18.8 | -9.9 | 28.6 | 11.2 | -5.9 | 13.8 | -6.3 | 1065 | 146 | 56 | 30.4 | 404 | 174 | 342 | 186 |
| at_kalk (16) | 47.82 | 14.5 | 757.6 | 0.66 | 6.8 | 80.0 | 285 | 7049 | 21.2 | -6.9 | 28.1 | 15.9 | -2.3 | 16.3 | -2.8 | 1031 | 144 | 58 | 31.4 | 416 | 183 | 370 | 209 |
| at_trogener (15) | 46.45 | 14.5 | 855.8 | 0.66 | 6.8 | 84.0 | 285 | 7247 | 21.8 | -7.6 | 29.4 | 16.5 | -2.6 | 16.5 | -3.2 | 1278 | 145 | 63 | 25.0 | 428 | 194 | 428 | 208 |
| at_loib (14) | 46.46 | 14.3 | 1118.4 | 0.93 | 6.2 | 83.6 | 286 | 7197 | 21.1 | -8.1 | 29.3 | 10.7 | -3.1 | 15.9 | -3.7 | 1644 | 179 | 83 | 22.2 | 514 | 256 | 495 | 292 |
| in_cansiglio (13) | 46.07 | 12.4 | 1080.2 | 0.84 | 7.0 | 81.0 | 286 | 6928 | 21.5 | -6.9 | 28.4 | 7.8 | -1.9 | 16.5 | -2.4 | 1427 | 151 | 78 | 20.4 | 438 | 236 | 393 | 264 |
| is_umbra (12) | 41.81 | 16.0 | 750.2 | 0.47 | 11.9 | 43.4 | 207 | 5793 | 23.1 | 2.3 | 20.8 | 10.4 | 19.7 | 20.6 | 4.6 | 618 | 71 | 37 | 20.4 | 204 | 116 | 118 | 148 |
| is_alburni (11) | 40.50 | 15.4 | 1213.0 | 0.88 | 9.0 | 84.4 | 321 | 6055 | 23.3 | -2.9 | 26.2 | 3.5 | 17.0 | 17.9 | 1.2 | 942 | 127 | 27 | 41.0 | 370 | 89 | 96 | 276 |
| is_calabre (10) | 39.50 | 16.1 | 1077.6 | 1.13 | 11.0 | 49.0 | 230 | 5657 | 22.6 | 1.4 | 21.2 | 8.4 | 18.4 | 19.5 | 3.8 | 1089 | 152 | 25 | 46.8 | 441 | 86 | 96 | 354 |
| fr_pavin (9) | 45.50 | 2.8 | 1272.6 | 0.88 | 6.4 | 77.0 | 314 | 5769 | 20.2 | -4.3 | 24.4 | 7.5 | 1.3 | 14.7 | -1.0 | 1113 | 117 | 73 | 13.6 | 322 | 231 | 263 | 278 |
| fr_chadefour (8) | 45.54 | 2.9 | 1181.8 | 0.80 | 6.1 | 77.0 | 315 | 5749 | 19.9 | -4.5 | 24.3 | 7.3 | 1.1 | 14.5 | -1.2 | 1032 | 106 | 67 | 12.2 | 291 | 211 | 264 | 251 |
| fr_picherande (7) | 45.47 | 2.8 | 1200.2 | 1.14 | 6.8 | 77.0 | 314 | 5765 | 20.6 | -3.8 | 24.4 | 0.9 | 4.4 | 15.2 | -0.5 | 1354 | 137 | 94 | 13.0 | 396 | 290 | 317 | 358 |
| ne_irati (6) | 42.99 | -1.1 | 855.8 | 1.28 | 9.9 | 85.6 | 355 | 5343 | 23.2 | -0.9 | 24.1 | 4.4 | 17.5 | 17.7 | 3.0 | 1332 | 152 | 65 | 24.2 | 450 | 209 | 209 | 402 |
| ne_saja (5) | 43.11 | -4.3 | 866.2 | 0.88 | 11.1 | 69.0 | 345 | 4557 | 22.2 | 2.2 | 20.1 | 6.7 | 17.9 | 17.9 | 5.4 | 925 | 109 | 44 | 25.2 | 308 | 142 | 142 | 278 |
| ne_redes (4) | 43.11 | -5.2 | 1237.4 | 1.17 | 8.7 | 78.6 | 365 | 4717 | 20.7 | -0.8 | 21.5 | 4.0 | 15.7 | 15.7 | 2.7 | 1174 | 146 | 47 | 30.6 | 404 | 145 | 145 | 367 |
| ce_cantalojas (3) | 41.23 | -3.4 | 1541.6 | 0.64 | 7.1 | 95.0 | 341 | 6184 | 23.2 | -4.5 | 27.8 | 0.8 | 16.5 | 16.5 | -0.6 | 707 | 80 | 27 | 28.0 | 228 | 82 | 82 | 202 |
| ce_pedrosa (2) | 41.22 | -3.4 | 1604.4 | 0.64 | 8.0 | 95.0 | 340 | 6231 | 24.2 | -3.7 | 27.9 | 1.6 | 17.5 | 17.5 | 0.2 | 681 | 78 | 24 | 30.0 | 222 | 72 | 72 | 200 |
| ce_montejo (1) | 41.11 | -3.5 | 1335.2 | 0.54 | 9.4 | 95.0 | 334 | 6378 | 25.9 | -2.5 | 28.4 | 2.9 | 19.2 | 19.2 | 1.5 | 563 | 71 | 17 | 34.0 | 201 | 54 | 54 | 168 |

Sampling sites: 1) Sitio Natural de Interés Nacional del Hayedo de Montejo de la Sierra; 2) Hayedo La Pedrosa; 3) Parque Natural Sierra Norte de Guadalajara; 4) Parque Natural de Redes; 5) Parque Natural Saja-Besaya; 6) La Selva de Irati; 7) Picherande; 8) Chadefour Valley Nature Reserve; 9) Réserve naturelle nationale de Chastreix-Sancy; 10) Riserva Statale Serra Nicolino - Pian d'Albero; 11) Parco Nazionale del Cilento e Valle de Diano; 12) Foresta Umbra; 13) Foresta del Cansiglio; 14) Loibltal; 15) Trögener Klamm; 16) Nationalpark Kalkalpen; 17) Klenovsý Vepor (Klenovské vrchy); 18) Cigánka-Muránsky hrad (Muránska planina NP); 19) Rabia skala

(Poloniny NP); 20) Bjurkärrs Naturreservat; 21) Biskopstorps Naturreservat; 22) Ramlaklitten i Skogsbo Naturreservat; 23) Ödegärdet Naturreservat. Countries: at=Austria, ce= Central Spain, fr= France, in=Northern Italy, is=Southern Italy, ne=northern Spain, sk=Slovakia, sw=Sweden.

**Table S2: Functional trait values.** Functional trait values for the 203 lichen species found in the 23 studied beech forests across Europe. Mean values ± SE of specific thallus mass (STM) in mg dry mass/cm$^2$, water-holding capacity (WHC) in mg water/cm$^2$, and carbon-nitrogen ratio (C/N) are provided for the machrolichen species (n=42 species for STM and WHC; n=57 species for C/N). Abbreviations: 1) Growth form: C=crustose, SQ=squamulose, L=leprose, FBL=foliose broad lobed, FNL=foliose narrow lobed, FR=fruticose dorsiventral, FRF=fruticose filamentous; 2) Reproductive strategy: ASEX=asexual; SEX=sexual; ASEX+SEX=both reproductive strategies (asexual and sexual); 3) Photobiont type: CB=cyanobacteria, CHL=green algae, TR=*Trentepohlia.*

| Lichen species | Growth form | Reproductive strategy | Photobiont type | STM | WHC | C/N |
|---|---|---|---|---|---|---|
| *Acrocordia cavata* (Ach.) R.C. Harris | C | SEX | TR | | | |
| *Acrocordia gemmata* (Ach.) A. Massal. var. *gemmata* | C | SEX | TR | | | |
| *Agonimia allobata* (Stizenb.) P. James | C | ASEX+SEX | CHL | | | |
| *Agonimia octospora* Coppins & P. James | SQ | SEX | CHL | | | |
| *Agonimia tristicula* (Nyl.) Zahlbr. | SQ | SEX | CHL | | | |
| *Alyxoria varia* (Pers.) Ertz & Tehler | C | SEX | TR | | | |
| *Amandinea punctata* (Hoffm.) Coppins & Scheid. | C | SEX | CHL | | | |
| *Anaptychia ciliaris* (L.) A. Massal. | FR | SEX | CHL | 12.58 ± 0.86 | 20.53 ± 1.32 | 51.6 ± 3.2 |
| *Anisomeridium biforme* (Schaer.) R.C. Harris | C | SEX | TR | | | |
| *Anisomeridium polypori* (Ellis & Everh.) M.E. Barr | C | SEX | TR | | | |
| *Arthonia atra* (Pers.) A. Schneid. | C | SEX | TR | | | |
| *Arthonia didyma* Körb. | C | SEX | TR | | | |
| *Arthonia punctiformis* Ach. | C | SEX | TR | | | |
| *Arthonia radiata* (Pers.) Ach. | C | SEX | TR | | | |
| *Arthonia* sp. | C | SEX | TR | | | |
| *Arthonia spadicea* Leight. | C | SEX | TR | | | |
| *Arthonia vinosa* Leight. | C | SEX | TR | | | |
| *Bacidia circumspecta* (Vain.) Malme | C | SEX | CHL | | | |
| *Bacidia incompta* (Borrer) Anzi | C | SEX | CHL | | | |
| *Bacidia laurocerasi* (Duby) Zahlbr. | C | SEX | CHL | | | |
| *Bacidia rosella* (Pers.) De Not. | C | SEX | CHL | | | |
| *Bacidia rubella* (Hoffm.) A. Massal. | C | SEX | CHL | | | |
| *Bacidia* sp. | C | SEX | CHL | | | |
| *Bacidia subincompta* (Nyl.) Arnold | C | ASEX+SEX | CHL | | | |
| *Bacidina arnoldiana* (Körb.) V. Wirth & Vězda | C | ASEX+SEX | CHL | | | |
| *Bacidina delicata* (Leight.) V. Wirth & Vězda | C | SEX | CHL | | | |
| *Biatora chrysantha* (Zahlbr.) Printzen | C | ASEX+SEX | CHL | | | |
| *Biatora efflorescens* (Hedl.) Räsänen | C | ASEX+SEX | CHL | | | |
| *Biatora vernalis* (L.) Fr. | C | SEX | CHL | | | |
| *Blastenia herbidella* (Hue) Servít | C | ASEX+SEX | CHL | | | |
| *Bryobilimbia hypnorum* (Lib.) Fryday, Printzen & S. Ekman | C | SEX | CHL | | | |
| *Bryoria fuscescens* (Gyeln.) Brodo & D. Hawksw. | FRF | ASEX | CHL | | | 29.4 ± 1.4 |
| *Buellia disciformis* (Fr.) Mudd | C | SEX | CHL | | | |
| *Buellia griseovirens* (Sm.) Almb. | C | ASEX | CHL | | | |

| Lichen species | Growth form | Reproductive strategy | Photobiont type | STM | WHC | C/N |
|---|---|---|---|---|---|---|

| | | | | | | |
|---|---|---|---|---|---|---|
| *Calicium salicinum* Pers. | C | SEX | CHL | | | |
| *Calicium viride* Pers. | C | SEX | CHL | | | |
| *Caloplaca obscurella* (J. Lahm) Th. Fr. | C | ASEX | CHL | | | |
| *Candelaria concolor* (Dicks.) Stein | FNL | ASEX+SEX | CHL | | | |
| *Candelariella vitellina* (Hoffm.) Müll. Arg. | C | SEX | CHL | | | |
| *Candelariella xanthostigma* (Ach.) Lettau | C | ASEX+SEX | CHL | | | |
| *Carbonicola myrmecina* (Ach.) Bendiksby & Timdal | SQ | ASEX+SEX | CHL | | | |
| *Catillaria nigroclavata* (Nyl.) J. Steiner | C | SEX | CHL | | | |
| *Cetrelia olivetorum* (Nyl.) W.L. Culb. & C.F. Culb. | FBL | ASEX+SEX | CHL | 6.51 ± 0.4 | 10.79 ± 0.72 | 42.3 ± 1.7 |
| *Chaenotheca furfuracea* (L.) Tibell | C | SEX | CHL | | | |
| *Chrysothrix candelaris* (L.) J.R. Laundon | L | ASEX+SEX | CHL | | | |
| *Cladonia chlorophaea* (Sommerf.) Spreng. | FR | ASEX+SEX | CHL | | | 41.4 ± 1.7 |
| *Cladonia coniocraea* (Flörke) Spreng. | FR | ASEX+SEX | CHL | | | 42.0 ± 1.8 |
| *Cladonia cornuta* (L.) Hoffm. | FR | ASEX+SEX | CHL | | | |
| *Cladonia digitata* (L.) Hoffm. | FR | ASEX+SEX | CHL | | | 35.1 ± 2.7 |
| *Cladonia fimbriata* (L.) Fr. | FR | ASEX+SEX | CHL | | | 43.5 ± 2.0 |
| *Cladonia parasitica* (Hoffm.) Hoffm. | FR | ASEX+SEX | CHL | | | |
| *Cladonia pyxidata* (L.) Hoffm. | FR | ASEX+SEX | CHL | | | 46.6 ± 1.7 |
| *Coenogonium luteum* (Dicks.) Kalb & Lücking | C | SEX | TR | | | |
| *Coenogonium pineti* (Ach.) Lücking & Lumbsch | C | SEX | TR | | | |
| *Collema flaccidum* (Ach.) Ach. | FBL | ASEX+SEX | CB | 3.3 ± 0.08 | 14.13 ± 0.39 | 10.2 ± 0.1 |
| *Collema furfuraceum* Du Rietz | FBL | ASEX+SEX | CB | 2.42 | 11.32 | 10.8 ± 0.2 |
| *Collema nigrescens* (Huds.) DC. | FBL | ASEX+SEX | CB | | | |
| *Collema subflaccidum* Degel. | FBL | ASEX+SEX | CB | | | |
| *Collema subnigrescens* Degel. | FBL | SEX | CB | 6.48 ± 0.72 | 89.84 ± 42.75 | 14.3 ± 2.2 |
| *Coniocarpon cinnabarinum* DC. | C | SEX | TR | | | |
| *Enterographa crassa* (DC.) Fée | C | SEX | TR | | | |
| *Evernia prunastri* (L.) Ach. | FR | ASEX+SEX | CHL | 9.15 ± 0.19 | 14.37 ± 0.24 | 45.4 ± 1.4 |
| *Flavoparmelia caperata* (L.) Hale | FBL | ASEX+SEX | CHL | 9.1 ± 0.47 | 10.76 ± 0.47 | 41.9 ± 2.8 |
| *Fuscidea stiriaca* (A. Massal.) Hafellner | C | SEX | CHL | | | |
| *Fuscopannaria leucosticta* (Tuck.) P.M. Jørg. | SQ | SEX | CB | | | |
| *Graphis elegans* (Sm.) Ach. | C | SEX | TR | | | |
| *Graphis scripta* (L.) Ach. | C | SEX | TR | | | |
| *Gyalecta carneola* (Ach.) Hellb. | C | SEX | TR | | | |
| *Heterodermia japonica* (M. Satô) Swinscow & Krog | FNL | ASEX | CHL | | | |
| *Heterodermia obscurata* (Nyl.) Trevis. | FNL | ASEX+SEX | CHL | | | |
| Lichen species | Growth form | Reproductive strategy | Photobiont type | STM | WHC | C/N |

| Lichen species | Growth form | Reproductive strategy | Photobiont type | STM | WHC | C/N |
|---|---|---|---|---|---|---|
| *Heterodermia speciosa* (Wulfen) Trevis. | FNL | ASEX+SEX | CHL | 18.38 ± 3.08 | 23.28 ± 3.63 | 45.8 ± 4.2 |
| *Hyperphyscia adglutinata* (Flörke) H. Mayrhofer & Poelt | FNL | ASEX+SEX | CHL | | | |
| *Hypogymnia farinacea* Zopf | FNL | ASEX+SEX | CHL | | | |
| *Hypogymnia physodes* (L.) Nyl. | FNL | ASEX+SEX | CHL | 9.05 ± 0.35 | 16.59 ± 0.49 | 46.4 ± 1.9 |
| *Hypogymnia tubulosa* (Schaer.) Hav. | FNL | ASEX+SEX | CHL | | | 44.3 ± 2 |
| *Lecania naegelii* (Hepp) Diederich & van den Boom | C | SEX | CHL | | | |
| *Lecanora albella* (Pers.) Ach. | C | SEX | CHL | | | |
| *Lecanora allophana* (Ach.) Nyl. f. *allophana* | C | SEX | CHL | | | |
| *Lecanora argentata* (Ach.) Malme | C | SEX | CHL | | | |
| *Lecanora carpinea* (L.) Vain. | C | SEX | CHL | | | |
| *Lecanora chlarotera* Nyl. subsp. *chlarotera* | C | SEX | CHL | | | |
| *Lecanora expallens* Ach. | C | ASEX+SEX | CHL | | | |
| *Lecanora glabrata* (Ach.) Nyl. | C | SEX | CHL | | | |
| *Lecanora horiza* (Ach.) Linds. | C | SEX | CHL | | | |
| *Lecanora intumescens* (Rebent.) Rabenh. | C | SEX | CHL | | | |
| *Lecanora leptyrodes* (Nyl.) Degel. | C | SEX | CHL | | | |
| *Lecanora pulicaris* (Pers.) Ach. | C | SEX | CHL | | | |
| *Lecidella elaeochroma* (Ach.) M. Choisy var. *elaeochroma* f. *elaeochroma* | C | SEX | CHL | | | |
| *Lecidella* sp. | C | SEX | CHL | | | |
| *Lepra albescens* (Huds.) Hafellner | C | ASEX | CHL | | | |
| *Lepra amara* (Ach.) Hafellner | C | ASEX | CHL | | | |
| *Lepra multipuncta* (Turner) Hafellner | C | ASEX+SEX | CHL | | | |
| *Lepraria incana* (L.) Ach. | L | ASEX | CHL | | | |
| *Lepraria membranacea* (Dicks.) Vain. | L | ASEX | CHL | | | |
| *Leptogium saturninum* (Dicks.) Nyl. | FBL | ASEX+SEX | CB | 5.69 ± 0.28 | 27.12 ± 2.63 | 14.3 ± 4.5 |
| *Lobaria pulmonaria* (L.) Hoffm. | FBL | ASEX+SEX | CHL | 11.9 ± 0.3 | 20.84 ± 0.56 | 20.5 ± 0.4 |
| *Lobarina scrobiculata* (Scop.) Nyl. | FBL | ASEX+SEX | CB | 12.05 ± 0.47 | 24.01 ± 0.89 | 16.2 ± 0.2 |
| *Loxospora elatina* (Ach.) A. Massal. | C | ASEX | CHL | | | |
| *Melanelixia fuliginosa* (Duby) O. Blanco, A. Crespo, Divakar, Essl., D. Hawksw. & Lumbsch | FNL | ASEX | CHL | 8.94 ± 0.33 | 15.42 ± 0.61 | 31.3 ± 1.5 |
| *Melanelixia glabra* (Schaer.) O. Blanco, A. Crespo, Divakar, Essl., D. Hawksw. & Lumbsch | FBL | SEX | CHL | 22.46 | 38.58 | 49.4 ± 3.2 |
| *Melanelixia subaurifera* (Nyl.) O. Blanco, A. Crespo, Divakar, Essl., D. Hawksw. & Lumbsch | FBL | ASEX+SEX | CHL | 6.75 ± 0.57 | 13.36 ± 0.92 | 30.1 ± 2.2 |
| *Melanohalea elegantula* (Zahlbr.) O. Blanco, A. Crespo, Divakar, Essl., D. Hawksw. & Lumbsch | FBL | ASEX+SEX | CHL | | | 37.1 ± 1.3 |
| *Melanohalea exasperatula* (Nyl.) O. Blanco, A. Crespo, Divakar, Essl., D. Hawksw. & Lumbsch | FBL | ASEX+SEX | CHL | 6.55 | 14.17 | 21.8 ± 2.3 |
| *Menegazzia terebrata* (Hoffm.) A. Massal. | FBL | ASEX+SEX | CHL | 13.23 ± 0.64 | 24.65 ± 1.62 | 55.5 ± 3.9 |

| | | | | | | |
|---|---|---|---|---|---|---|
| *Micarea adnata* Coppins | C | SEX | CHL | | | |
| *Micarea denigrata* (Fr.) Hedl. | C | SEX | CHL | | | |
| *Micarea peliocarpa* (Anzi) Coppins & R. Sant. | C | SEX | CHL | | | |
| *Micarea prasina* Fr. | C | SEX | CHL | | | |
| *Mycobilimbia carneoalbida* (Müll. Arg.) S. Ekman & Printzen | C | SEX | CHL | | | |
| *Mycobilimbia pilularis* (Körb.) Hafellner & Türk | C | SEX | CHL | | | |
| *Myriolecis albescens* (Hoffm.) Sliwa, Zhao Xin & Lumbsch | C | SEX | CHL | | | |
| *Myriolecis hagenii* (Ach.) Sliwa, Zhao Xin & Lumbsch | C | SEX | CHL | | | |
| *Nephroma laevigatum* Ach. | FBL | SEX | CB | 5.7 ± 0.21 | 12.96 ± 0.45 | 17.9 ± 2.5 |
| *Nephroma parile* (Ach.) Ach. | FBL | ASEX+SEX | CB | 6.39 ± 0.38 | 14.71 ± 0.71 | 11.7 ± 0.2 |
| *Nephroma resupinatum* (L.) Ach. | FBL | SEX | CB | 6.49 ± 0.18 | 18.03 ± 0.44 | 14.7 ± 1.8 |
| *Nevesia sampaiana* (Tav.) P.M. Jørg., L. Lindblom, Wedin & S. Ekman | SQ | ASEX | CB | | | 9.9 ± 0.1 |
| *Normandina pulchella* (Borrer) Nyl. | SQ | ASEX+SEX | CHL | | | |
| *Ochrolechia balcanica* Verseghy | C | SEX | CHL | | | |
| *Ochrolechia pallescens* (L.) A. Massal. | C | SEX | CHL | | | |
| *Ochrolechia subviridis* (Høeg) Erichsen | C | ASEX | CHL | | | |
| *Ochrolechia szatalaensis* Verseghy | C | SEX | CHL | | | |
| *Ochrolechia turneri* (Sm.) Hasselrot | C | ASEX+SEX | CHL | | | |
| *Opegrapha* sp. | C | SEX | TR | | | |
| *Opegrapha trochodes* Coppins, F. Berger & Ertz | C | SEX | TR | | | |
| *Opegrapha vermicellifera* (Kunze) J.R. Laundon | C | SEX | TR | | | |
| *Pannaria conoplea* (Ach.) Bory | FNL | ASEX+SEX | CB | | | 18.6 ± 5.7 |
| *Pannaria rubiginosa* (Ach.) Bory | FNL | SEX | CB | | | |
| *Pannaria tavaresii* P.M. Jørg. | SQ | ASEX+SEX | CB | | | |
| *Parmelia saxatilis* (L.) Ach. | FBL | ASEX+SEX | CHL | 13.54 ± 0.46 | 20.32 ± 0.76 | 41.4 ± 1.1 |
| *Parmelia submontana* Hale | FBL | ASEX+SEX | CHL | 8.0 ± 0.34 | 11.67 ± 0.43 | 37.4 ± 2.9 |
| *Parmelia sulcata* Taylor | FBL | ASEX+SEX | CHL | 10.93 ± 0.26 | 17.47 ± 0.47 | 45.8 ± 1.3 |
| *Parmeliella triptophylla* (Ach.) Müll. Arg. | C | ASEX+SEX | CB | | | 11.3 ± 0.7 |
| *Parmelina pastillifera* (Harm.) Hale | FBL | ASEX+SEX | CHL | 9.55 ± 0.44 | 14.34 ± 0.89 | 26.9 ± 4.5 |
| *Parmelina tiliacea* (Hoffm.) Hale | FBL | ASEX+SEX | CHL | 7.93 ± 0.24 | 12.96 ± 0.38 | 32 ± 1.4 |
| *Parmeliopsis ambigua* (Hoffm.) Nyl. | FNL | ASEX+SEX | CHL | | | |
| *Parmeliopsis hyperopta* (Ach.) Arnold | FNL | ASEX+SEX | CHL | | | |
| *Parmotrema perlatum* (Huds.) M. Choisy | FBL | ASEX+SEX | CHL | 6.17 ± 0.26 | 9.31 ± 0.29 | 41.7 ± 4.9 |
| Lichen species | Growth form | Reproductive strategy | Photobiont type | STM | WHC | C/N |

| | | | | | | |
|---|---|---|---|---|---|---|
| *Pectenia plumbea* (Lightf.) P.M. Jørg., L. Lindblom, Wedin & S. Ekman | FNL | SEX | CB | 32.29 ± 4.24 | 116.9 ± 17.38 | 10 ± 0.6 |
| *Peltigera collina* (Ach.) Schrad. | FBL | ASEX+SEX | CB | 7.99 ± 0.3 | 21.58 ± 0.7 | 11.7 ± 0.2 |
| *Peltigera degenii* Gyeln. | FBL | ASEX+SEX | CB | | | 9.6 ± 0.3 |
| *Peltigera horizontalis* (Huds.) Baumg. | FBL | SEX | CB | 7.87 ± 0.26 | 23.33 ± 0.47 | 11.3 ± 0.3 |
| *Peltigera membranacea* (Ach.) Nyl. | FBL | SEX | CB | 10.42 | 27.1 | 10.6 ± 0.2 |
| *Peltigera praetextata* (Sommerf.) Zopf | FBL | ASEX+SEX | CB | 8.22 ± 0.25 | 22.59 ± 0.62 | 10.2 ± 0.1 |
| *Pertusaria coccodes* (Ach.) Nyl. | C | ASEX+SEX | CHL | | | |
| *Pertusaria coronata* (Ach.) Th. Fr. | C | ASEX+SEX | CHL | | | |
| *Pertusaria flavida* (DC.) J.R. Laundon | C | ASEX | CHL | | | |
| *Pertusaria hymenea* (Ach.) Schaer. | C | SEX | CHL | | | |
| *Pertusaria leioplaca* (Ach.) DC. | C | SEX | CHL | | | |
| *Pertusaria pertusa* (L.) Tuck. var. *pertusa* | C | SEX | CHL | | | |
| *Pertusaria pupillaris* (Nyl.) Th. Fr. | C | ASEX+SEX | CHL | | | |
| *Phaeographis lyellii* (Sm.) Zahlbr. | C | SEX | TR | | | |
| *Phaeophyscia endophoenicea* (Harm.) Moberg | FNL | ASEX | CHL | | | |
| *Phaeophyscia orbicularis* (Neck.) Moberg | FNL | ASEX+SEX | CHL | | | |
| *Phlyctis agelaea* (Ach.) Flot. | C | ASEX+SEX | CHL | | | |
| *Phlyctis argena* (Spreng.) Flot. | C | ASEX+SEX | CHL | | | |
| *Physcia adscendens* H. Olivier | FNL | ASEX+SEX | CHL | | | |
| *Physcia aipolia* (Humb.) Fürnr. | FNL | SEX | CHL | | | |
| *Physcia leptalea* (Ach.) DC. | FNL | SEX | CHL | | | |
| *Physcia tenella* (Scop.) DC. | FNL | ASEX+SEX | CHL | | | |
| *Physconia distorta* (With.) J.R. Laundon | FNL | SEX | CHL | 17.7 ± 0.9 | 27.69 ± 2.33 | 31.9 ± 0.9 |
| *Physconia perisidiosa* (Erichsen) Moberg | FNL | ASEX+SEX | CHL | 13.89 ± 2.62 | 29.22 ± 7.95 | 27.7 ± 1.6 |
| *Physconia venusta* (Ach.) Poelt | FNL | SEX | CHL | 16.78 ± 1.4 | 31.61 ± 3.96 | 27.9 ± 1.6 |
| *Platismatia glauca* (L.) W.L. Culb. & C.F. Culb. | FBL | ASEX+SEX | CHL | 7 ± 0.17 | 11.37 ± 0.3 | 47 ± 1.4 |
| *Pleurosticta acetabulum* (Neck.) Elix & Lumbsch | FBL | SEX | CHL | | | 40.1 ± 3.4 |
| *Porina aenea* (Wallr.) Zahlbr. | C | SEX | TR | | | |
| *Porina hibernica* P. James & Swinscow | C | SEX | TR | | | |
| *Protopannaria pezizoides* (Weber) P.M. Jørg. & S. Ekman | C | SEX | CB | | | |
| *Pseudevernia furfuracea* (L.) Zopf var. *furfuracea* | FBL | ASEX+SEX | CHL | 8.4 ± 0.4 | 14.44 ± 0.57 | 32.9 ± 1.4 |
| *Psilolechia lucida* (Ach.) M. Choisy | L | SEX | CHL | | | |
| *Psoroglaena stigonemoides* (Orange) Henssen | C | ASEX+SEX | CHL | | | |
| *Pyrenula macrospora* (Degel.) Coppins & P. James | C | SEX | TR | | | |
| Lichen species | Growth form | Reproductive strategy | Photobiont type | STM | WHC | C/N |
| *Pyrenula nitida* (Weigel) Ach. | C | SEX | TR | | | |

| | | | | | | |
|---|---|---|---|---|---|---|
| *Pyrenula nitidella* (Schaer.) Müll. Arg. | C | SEX | TR | | | |
| *Pyrrhospora quernea* (Dicks.) Körb. | C | ASEX+SEX | CHL | | | |
| *Ramalina canariensis* J. Steiner | FR | ASEX+SEX | CHL | | | |
| *Ramalina farinacea* (L.) Ach. | FR | ASEX+SEX | CHL | 11.33 ± 0.37 | 15.43 ± 0.55 | 41.6 ± 1.9 |
| *Ramalina fastigiata* (Pers.) Ach. | FR | SEX | CHL | 12.49 ± 0.49 | 18.84 ± 0.74 | 39.9 ± 2.2 |
| *Ramalina fraxinea* (L.) Ach. | FR | SEX | CHL | 15.88 ± 1.66 | 26.35 ± 3.09 | 42.3 ± 2.4 |
| *Ramalina pollinaria* (Westr.) Ach. | FR | ASEX+SEX | CHL | | | |
| *Ricasolia amplissima* (Scop.) De Not. | FBL | SEX | CHL | 17.67 ± 0.59 | 29.63 ± 0.98 | 20.1 ± 0.6 |
| *Ricasolia virens* (With.) H.H. Blom. & Tønsberg | FBL | SEX | CHL | | | 17.2 ± 0.7 |
| *Rinodina colobina* (Ach.) Th. Fr. | C | ASEX+SEX | CHL | | | |
| *Rinodina griseosoralifera* Coppins | C | ASEX+SEX | CHL | | | |
| *Rinodina pyrina* (Ach.) Arnold | C | SEX | CHL | | | |
| *Scoliciosporum umbrinum* (Ach.) Arnold | C | SEX | CHL | | | |
| *Scytinium aragonii* (Otálora) Otálora, P.M. Jørg. & Wedin | SQ | SEX | CB | | | |
| *Scytinium lichenoides* (L.) Otálora, P.M. Jørg. & Wedin | SQ | ASEX+SEX | CB | | | |
| Sorediate | C | ASEX | CHL | | | |
| *Sphaerophorus globosus* (Huds.) Vain. | FR | SEX | CHL | | | 60.8 ± 4.7 |
| *Sticta limbata* (Sm.) Ach. | FBL | ASEX+SEX | CB | 7.77 ± 0.46 | 21.57 ± 1.81 | 10 ± 0.2 |
| *Tephromela atra* (Huds.) Hafellner var. *atra* | C | SEX | CHL | | | |
| *Thelenella muscorum* (Th. Fr.) Vain. var. *muscorum* | C | SEX | CHL | | | |
| *Thelopsis rubella* Nyl. | C | SEX | TR | | | |
| *Thelotrema lepadinum* (Ach.) Ach. | C | SEX | TR | | | |
| *Trapeliopsis flexuosa* (Fr.) Coppins & P. James | C | ASEX | CHL | | | |
| *Trapeliopsis gelatinosa* (Flörke) Coppins & P. James | C | ASEX | CHL | | | |
| *Usnea hirta* (L.) F.H. Wigg. | FRF | ASEX+SEX | CHL | | | |
| *Usnea longissima* Ach. | FRF | ASEX+SEX | CHL | | | |
| *Usnea subfloridana* Stirt. | FRF | ASEX+SEX | CHL | 12.06 ± 0.95 | 17.22 ± 1.32 | 34.8 ± 2 |
| *Varicellaria hemisphaerica* (Flörke) I. Schmitt & Lumbsch | C | ASEX | CHL | | | |
| *Vulpicida pinastri* (Scop.) J.-E. Mattsson & M.J. Lai | FBL | ASEX+SEX | CHL | | | |
| *Xanthoria parietina* (L.) Th. Fr. | FBL | SEX | CHL | | | |
| *Zwackhia viridis* (Ach.) Poetsch & Schied. | C | SEX | TR | | | |

**Figure S1: Phylogenetic tree.** Phylogenetic tree based on four molecular markers (nuITS, nuLSU, mtSSU and RPB1) including the lichen species found across Europe. Numbers above nodes denote the bootstrap support (ML-BS) obtained with Maximum Likelihood in RAxML. Species names can be found in Table S2.

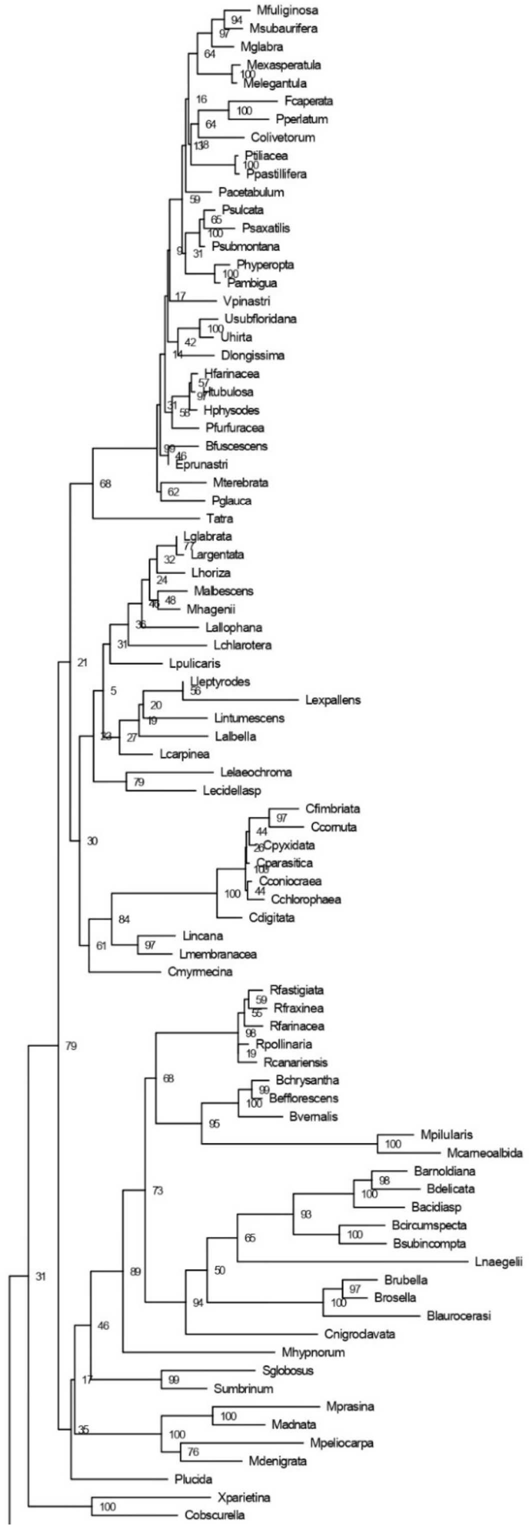

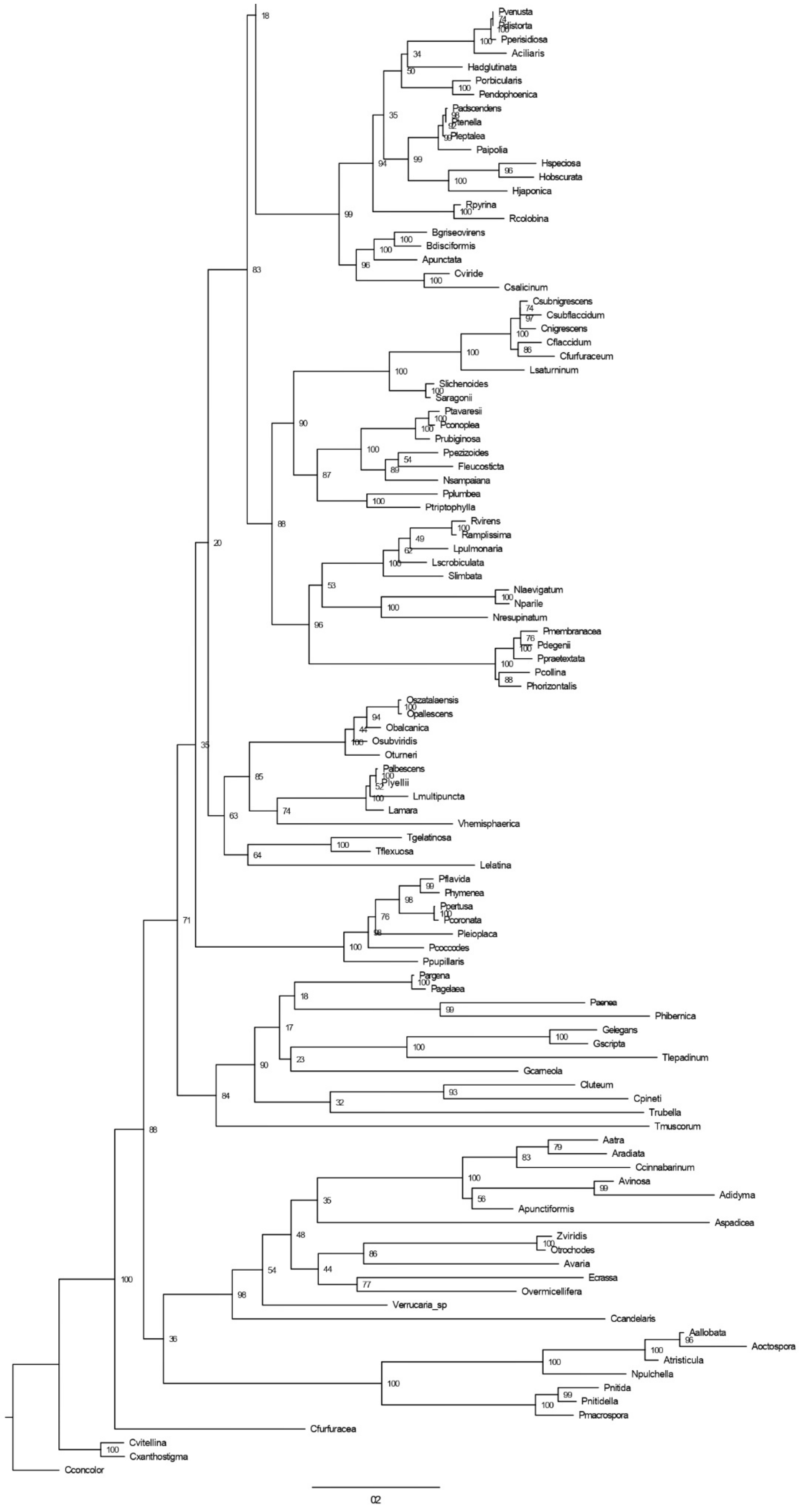
Pvenusta
Pdistorta
Pperisidiosa
Aciliaris
Hadglutinata
Porbicularis
Pendophoenica
Padscendens
Ptenella
Pleptalea
Paipolia
Hspeciosa
Hobscurata
Hjaponica
Rpyrina
Rcolobina
Bgriseovirens
Bdisciformis
Apunctata
Cviride
Csalicinum
Csubnigrescens
Csubflaccidum
Cnigrescens
Cflaccidum
Cfurfuraceum
Lsaturninum
Slichenoides
Saragonii
Ptavaresii
Pconoplea
Prubiginosa
Ppezizoides
Fleucosticta
Nsampaiana
Pplumbea
Ptriptophylla
Rvirens
Ramplissima
Lpulmonaria
Lscrobiculata
Slimbata
Nlaevigatum
Nparile
Nresupinatum
Pmembranacea
Pdegenii
Ppraetextata
Pcollina
Phorizontalis
Oszatalaensis
Opallescens
Obalcanica
Osubviridis
Oturneri
Palbescens
Plyellii
Lmultipuncta
Lamara
Vhemisphaerica
Tgelatinosa
Tflexuosa
Lelatina
Pflavida
Phymenea
Ppertusa
Pcoronata
Pleioplaca
Pcoccodes
Ppupillaris
Pargena
Pagelaea
Paenea
Phibernica
Gelegans
Gscripta
Tlepadinum
Gcarneola
Cluteum
Cpineti
Trubella
Tmuscorum
Aatra
Aradiata
Ccinnabarinum
Avinosa
Adidyma
Apunctiformis
Aspadicea
Zviridis
Otrochodes
Avaria
Ecrassa
Overmicellifera
Verrucaria_sp
Ccandelaris
Aallobata
Aoctospora
Atristicula
Npulchella
Pnitida
Pnitidella
Pmacrospora
Cfurfuracea
Cvitellina
Cxanthostigma
Cconcolor
02

**Table S3: Phylogenetic signal.** Phylogenetic signal of the three categorical (growth form, photobiont type and reproductive strategy) and three quantitative traits studied (specific thallus mass, STM; water-holding capacity, WHC; and carbon-nitrogen ratio C/N).

| | Trait | Nº of levels | Observed transitions | Median null model (p-value) | Pagel's Lambda (λ) |
|---|---|---|---|---|---|
| Qualitative | Growth form | 7 | 29 | 86*** | - |
| | Photobiont type | 3 | 10 | 51*** | - |
| | Reproductive strategy | 3 | 49 | 75*** | - |
| Quantitative | STM | - | - | - | 0.95 |
| | WHC | - | - | - | 0.80 |
| | C/N | | | | 0.87 |

No. of levels: number of categories for a given categorical trait; Observed transitions: number of observed evolutionary transitions; Median Null Model: median of expected evolutionary transitions under a null model in which the tips of the phylogeny were randomised 1,000 times; p-value based on the comparison of observed and expected evolutionary transitions (***$p < 0.001$).

**Supplementary 4: Diversity metrics**

Regarding the functional characterization of the epiphytic communities, we used individual species trait data to calculate two indices at community level: community weighted mean (hereafter 'CWM') and Rao's quadratic entropy index (hereafter 'Rao'). These two indices inform about different components of functional diversity: while CWM reflects the dominant traits in a community, Rao is a multivariate form of the functional variance [1]. As an indicator of functional composition, we calculated the CWM index of every qualitative trait for each of the forests studied using the *functcomp* function implemented in the *FD* package [2]. This index was computed as the mean trait value of each species in a community, weighted by the relative abundance of this species [3]. The larger the relative abundance of one species, the more important contribution of its individual trait value to the global community average. For qualitative traits, the CWM reflects the percentage of a given category of the trait in a community. As an indicator of functional variance, we calculated a Rao index considering multiple qualitative and quantitative traits together (i.e. growth form, photobiont type, reproductive strategy, STM, WHC and C/N). First, we calculated the species dissimilarity matrix using Gower distances and giving more weight to quantitative traits (w = 1) than qualitative traits (w = 0.5) since the latter usually show higher values of dissimilarity. Dissimilarity distances ($d_{ij}$) closer to 0 denote that species are functionally equivalent, while $d_{ij}$ values closer to 1 reflect higher dissimilarity between species [4]. Then, using the trait dissimilarity distances between each pair of coexisting species ($d_{ij}$) and the relative abundance of these species in a given forest ($p_i$ and $p_j$), we computed the Rao index at forest level with the function *Rao* [1]:

$$FD_{Rao} = \sum_{i=1}^{S} \cdot \sum_{j=1}^{S} \cdot p_i p_j d_{ij}$$

We finally applied the Jost correction [5] to express the index in equivalent numbers. Higher values of Rao index denote higher community functional diversity in a given forest. Some of the advantages of this metric are the combination of functional richness and functional divergence [6], and the quantification of species dissimilarity including the relative abundance of species [1].

Since Rao allows the measurement of species dissimilarity based on functional and phylogenetic data, it is a useful metric to compare these different diversity facets (i.e. FD and PD) [1]. Hence, we calculated a Rao index combining the species relative abundance with the phylogenetic tree as metric of PD. In this case, we used the function *cophenetic* implemented in the *picante* package [7], to compute the phylogenetic distance between pairs of coexisting species (*dij*).

## References


1. de Bello, F.; Lavergne, S.; Meynard, C.N.; Lepš, J.; Thuiller, W. The partitioning of diversity: Showing Theseus a way out of the labyrinth. *J. Veg. Sci.* ***2010***, *21*, 992–1000. DOI: 10.1111/j.1654-1103.2010.01195.x
2. Laliberté, E.; Legendre, P. A distance-based framework for measuring functional diversity from multiple traits. *Ecology* **2010,** *91*, 299–305. DOI: 10.1890/08-2244.1
3. Lavorel, S.; Grigulis, K.; McIntyre, S.; Williams, N.S.G.; Garden, D.; Dorrough, J.; Berman, S.; Quetier, F.; Thébault, A.; Bonis, A. Assessing functional diversity in the field - Methodology matters! *Funct. Ecol.* **2008,** *22*, 134–147. DOI: 10.1111/j.1365-2435.2007.01339.x
4. Pavoine, S.; Vallet, J.; Dufour, A.B.; Gachet, S.; Daniel, H. On the challenge of treating various types of variables: Application for improving the measurement of functional diversity. *Oikos* **2009,** *118*, 391–402. DOI: 10.1111/j.1600-0706.2008.16668.x
5. Jost, L. Partitioning diversity into independent alpha and beta components. *Ecology* **2007,** *88*, 2427–2439. DOI: 10.1890/06-1736.1
6. Mouchet, M.A.; Villéger, S.; Mason, N.W.H.; Mouillot, D. Functional diversity measures: An overview of their redundancy and their ability to discriminate community assembly rules. *Funct. Ecol.* **2010,** *24*, 867–876. DOI: 10.1111/j.1365-2435.2010.01695.x
7. Kembel, S.W.; Cowan, P.D.; Helmus, M.R.; Cornwell, W.K.; Morlon, H.; Ackerly, D.D.; Blomberg, S.P.; Webb, C.O. Picante: R tools for integrating phylogenies and ecology. *Bioinformatics* **2010,** *26*, 1463–1464. DOI: 10.1093/bioinformatics/btq166

**Figure S2: Relationships among TD, FD, and PD.** Scatterplots representing the relation between different diversity metrics for the 23 beech forests surveyed across Europe. 1) taxonomic diversity, TD (Shannon and Inverse Simpson) and functional diversity, FD (Rao); 2) phylogenetic diversity, PD (Rao) and FD (Rao); and 3) evenness (Shannon/ln(richness)) and FD (Rao).

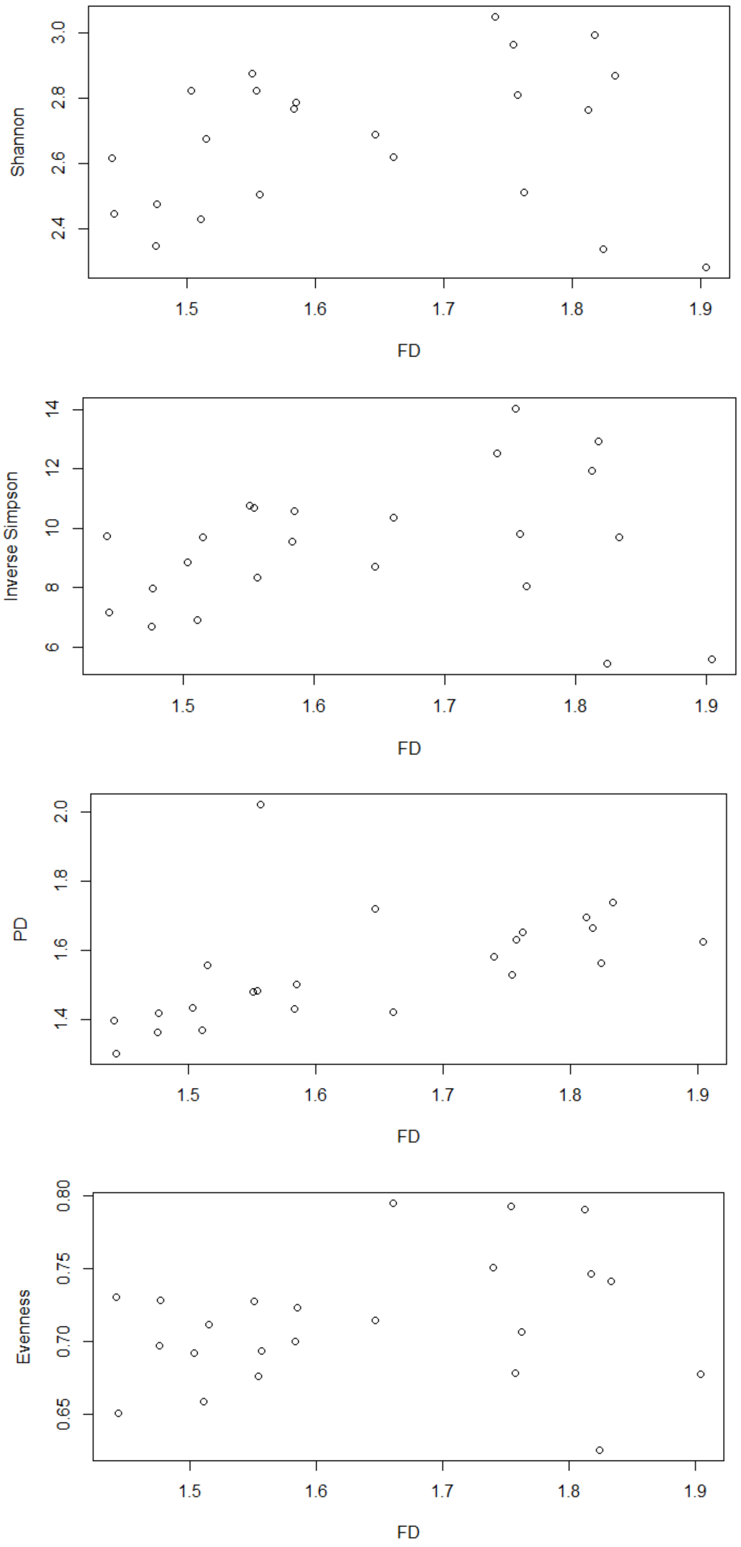

**Table S4: Ranking of linear models of species richness, Shannon, Inverse Simpson, Rao FD (multitrait) and Rao PD following an AIC-based model selection procedure (ΔAICc ≤ 2).** For each community diversity metric, the best model (i.e. lowest AICc value) is presented in the first row followed by the rest of models with ΔAICc ≤ 2. Columns represent environmental predictors related to forest structure (DBH) and climate (temperature and precipitation). Grey cells indicate environmental predictors included in a particular model. $R^2$, percentage of variance explained by a particular model; AICc, relative goodness of fit; ΔAICc, AIC differences; wi, Akaike weight; $I_P$, Moran index p-value. Abbreviations: TD, taxonomic diversity; FD, functional diversity; PD, phylogenetic diversity; DBH, tree diameter at breast height; BIO2, Annual Mean Diurnal Range; BIO5, Max Temperature of the Warmest Month; BIO6, Min Temperature of the Coldest Month; BIO8, Mean Temperature of the Wettest Quarter; BIO13, Precipitation of the Wettest Month; BIO14, Precipitation of the Driest Month.

| | | ENVIRONMENTAL PREDICTORS | | | | | | | | | | | |
|---|---|---|---|---|---|---|---|---|---|---|---|---|---|
| | | | Temperature | | | | Precipitation | | $R^2$ | $AIC_c$ | $\Delta AIC_c$ | $w_i$ | $I_P$ |
| | | DBH | BIO2 | BIO5 | BIO6 | BIO8 | BIO13 | BIO14 | | | | | |
| TD | Richness | | | | ■ | ■ | ■ | | 0.59 | 168.2 | 0 | 0.67 | 0.82 |
| | | | | | ■ | ■ | ■ | ■ | 0.63 | 169.6 | 1.45 | 0.33 | 0.68 |
| | Shannon | | | | | | ■ | | 0.22 | -3.7 | 0 | 0.38 | 0.61 |
| | | | | ■ | | | ■ | | 0.29 | -2.8 | 0.9 | 0.24 | 0.96 |
| | | | | | ■ | | ■ | | 0.28 | -2.6 | 1.08 | 0.22 | 0.73 |
| | | | | | | | ■ | ■ | 0.26 | -1.9 | 1.79 | 0.15 | 0.78 |
| | Inverse Simpson | | | | | | ■ | | 0.13 | 105.6 | 0 | 0.46 | 0.57 |
| | | | | ■ | | | ■ | | 0.17 | 107.4 | 1.80 | 0.19 | 0.37 |
| FD | Rao functional | | ■ | | | | | | 0.27 | -24.1 | 0 | 1 | 0.07 |
| PD | Rao phylogenetic | | ■ | | ■ | | | | 0.25 | -16.2 | 0 | 0.15 | 0.30 |
| | | ■ | ■ | | | | | | 0.24 | -16.2 | 0.02 | 0.15 | 0.41 |
| | | | ■ | | | | | | 0.14 | -16.1 | 0.09 | 0.15 | 0.06 |
| | | ■ | | | | | | | 0.13 | -15.9 | 0.32 | 0.13 | 0.14 |
| | | ■ | ■ | | ■ | | | | 0.33 | -15.8 | 0.34 | 0.13 | 0.78 |
| | | ■ | | ■ | | | | | 0.21 | -15.1 | 1.10 | 0.09 | 0.21 |
| | | | | ■ | | | | | 0.09 | -14.8 | 1.34 | 0.08 | 0.02* |

**Figure S3: Variation partitioning Venn diagram for Inverse Simpson.** Variation partitioning Venn diagram representing the percentages of unique and shared contribution of climate (precipitation of the wettest month) and functional diversity (FD) to $TD_{Inverse\ Simpson}$ variation. The intersection represents the amount (%) of explained variation shared by different explanatory variables. Residuals represent the % of unexplained variation.

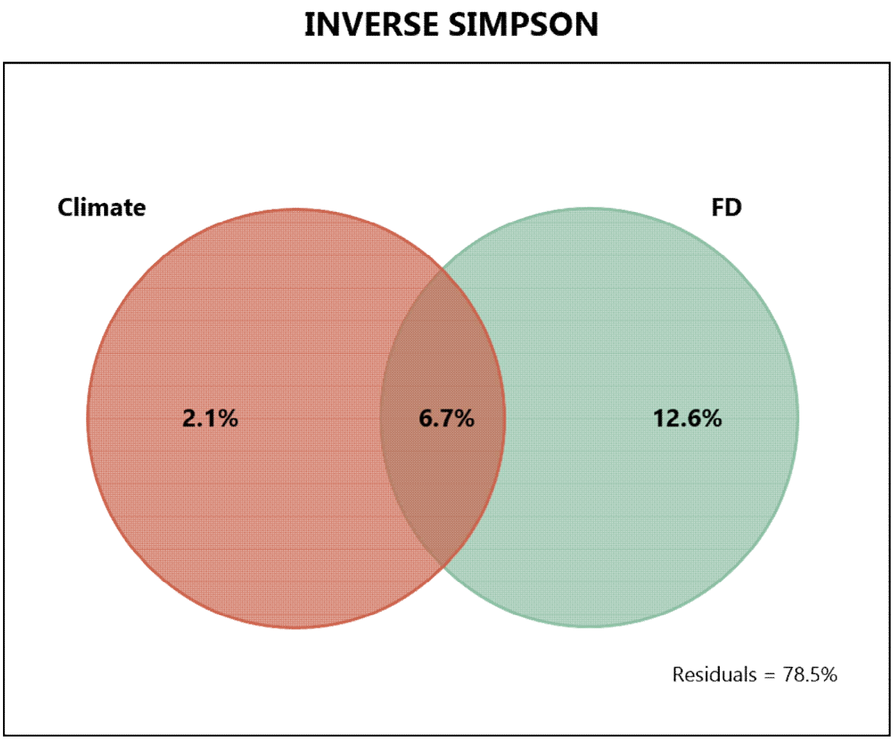


**Publisher's Note:** MDPI stays neutral with regard to jurisdictional claims in published maps and institutional affiliations.

© 2020 by the authors. Submitted for possible open access publication under the terms and conditions of the Creative Commons Attribution (CC BY) license (http://creativecommons.org/licenses/by/4.0/).
